# Does Medical Students' Preference of Test Format (Computer-based vs. Paper-based) have an Influence on Performance?

**DOI:** 10.1186/1472-6920-11-89

**Published:** 2011-10-25

**Authors:** Achim Hochlehnert, Konstantin Brass, Andreas Moeltner, Jana Juenger

**Affiliations:** 1 Department of Psychosomatic and General Internal Medicine, University of Heidelberg, Medical Clinic, Heidelberg, Germany; 2 Center of Excellence for Medical Assessment, University of Heidelberg, Medical School, Heidelberg, Germany

**Keywords:** computer-based examination, paper-based examination, usability

## Abstract

**Background:**

Computer-based examinations (CBE) ensure higher efficiency with respect to producibility and assessment compared to paper-based examinations (PBE). However, students often have objections against CBE and are afraid of getting poorer results in a CBE.

The aims of this study were (1) to assess the readiness and the objections of students to a CBE vs. PBE (2) to examine the acceptance and satisfaction with the CBE on a voluntary basis, and (3) to compare the results of the examinations, which were conducted in different formats.

**Methods:**

Fifth year medical students were introduced to an examination-player and were free to choose their format for the test. The reason behind the choice of the format as well as the satisfaction with the choice was evaluated after the test with a questionnaire. Additionally, the expected and achieved examination results were measured.

**Results:**

Out of 98 students, 36 voluntarily chose a CBE (37%), 62 students chose a PBE (63%). Both groups did not differ concerning sex, computer-experience, their achieved examination results of the test, and their satisfaction with the chosen format. Reasons for the students' objections against CBE include the possibility for outlines or written notices, a better overview, additional noise from the keyboard or missing habits normally present in a paper based exam. The students with the CBE tended to judge their examination to be more clear and understandable. Moreover, they saw their results to be independent of the format.

**Conclusions:**

Voluntary computer-based examinations lead to equal test scores compared to a paper-based format.

## Background

The use of computer-based examinations combines advantages with respect to content (integration of other media, favourable presentation of pictures, and possibility of other examination formats) with rapid data analysis. This promises higher efficiency with respect to implementation and evaluation [1-3]. However, students often have worries and prejudices concerning an unsatisfactory graphical user interface (GUI) of the examination software, possible technical problems with the computer, concentration problems, and additional exam stress [4]. An increase of the number of required graded examinations during medical studies from zero to 39 has been one effect of the new medical licensing regulations in Germany [5]. This presents a challenge to academic departments, especially those with limited teaching personnel and financial resources. To our knowledge this is the first study comparing voluntary computer-based examinations (CBE) and paper-based examinations (PBE). The aims of this study were (1) to assess the readiness of students to a CBE vs. PBE (2) to examine the acceptance and satisfaction with the CBE on a voluntary basis, and (3) to compare the results of the examinations, which were conducted in different formats.

## Methods

The examination in human genetics in the University of Heidelberg is usually performed paper-based. For our study 98 fifth-year medical students were offered a choice between CBE and PBE for a written human genetics exam after a two week course. All of the students were introduced to both options at the beginning of the course. Due to concerns of a possible disadvantage in a CBE, all students received a short introduction to the examination software (Campus player version 181205 [6]). Suggestions for improvement concerning possible disparities of the GUI compared to the PBE were implemented in a new update. Afterwards, the students were free to decide for an examination format. The test consisted of 26 questions, 24 multiple-choice-format with differing types (type A and Kprim) and two short answer questions. The test lasted 90 minutes in both examination formats. Both formats consisted of identical questions which could be answered in optional order. After the test, the students answered a questionnaire with 19 items to evaluate their acceptance and the reason for their choice as well as the usability of the software on a Likert scale from 1 to 5 (see table 1). Furthermore, questions concerning previous experience with CBE and opinions thereof were asked.

**Table 1:** Questionnaire

	Mean	StdDev	Mean	StdDev	Pr > |t|
	computer-based examination format (n = 36)		paper-based examination format (n = 62)		
1) The usability of the examination was easy.	4.556	0.843	4.667	0.636	0.5004
2) The examination was clear and easily understandable.	4.361	1.073	4.000	1.118	0.1240
3) Additional mental effort was required due to the chosen examination format.	2.306	1.390	1.123	0.381	**<.0001**
4) I found it useful to take the examination in the chosen format, because it increased efficacy in this situation.	2.972	0.941	4.375	0.822	**<.0001**
5) I was anxious before the examination.	3.556	1.054	3.789	1.048	0.2995
6) After a few questions, my anxiety at the beginning of the examination was gone.	3.528	1.055	3.211	1.264	0.1949
7) If I had a choice, I would take future examinations more often in the chosen format.	3.722	1.111	4.596	0.593	**<.0001**
8) Expected scoring in examination	18.72	2.387	17.15	3.664	**0.0218**
9) CBE: If I would have chosen PBE I suppose my results would have been (better/equal/worse). PBE: If I would have chosen CBE I suppose my results would have been (better/equal/worse).	0.086	0.284	-0.452	0.563	**<.0001**
10) In how many CBEs did you participate so far?	2.250	1.180	2.500	1.059	0.3016
11) It is an advantage of CBE that there is the possibility to change my answers during the examination.	4.371	0.770	3.344	1.263	**<.0001**
12) It is important for me to have the possibility to change my answers during the examination.	4.200	0.933	2.900	1.231	**<.0001**
13) I found it useful to take this examination in CBE-format because it prepared me for further upcoming CBEs. (only CBE)	3.657	1.259	-	-	-
14) The CBE itself was in total better than I expected it to be. (only CBE)	3.417	1.025	-	-	-
15) There were no technical problems in the CBE. (only CBE)	4.722	0.615	-	-	-
16) I was satisfied with the graphical user interface. (only CBE)	4.714	0.667	-	-	-
17) Reason for preference (open question)	-	-	-	-	-
18) suggestions for improvement (open question)	-	-	-	-	-
19) I decline CBE in general. (only PBE)	-	-	2.491	1.269	-

items 1-7 and 11-17: Likert scale with scoring from 'I completely agree' (5) to 'I do not agree at all' (1), items 8 and 10: numerical scale, item 9: +1 (better), 0 (equal), -1 (worse), items 18 and 19: open question. For statistical evaluation a t-test with Satterthwaite-correction for unequal variances was conducted.

For statistical evaluation a t-test with Satterthwaite-correction for unequal variances was conducted. The statistical evaluation was performed with SAS 9.1.

## Results

36 (37%) of the students chose the computer-based examination (CBE) and 62 (63%) chose the paper-based examination (PBE). The ratio of male to female students in both exam formats was similar (CBE: m = 14, f = 22; PBE: m = 26, f = 36; not statistically significant). On average, both groups had the same level of previous experience with CBE (CBP: 3.7; PBE: 3.5; not statistically significant), which is very little compared to the numerous PBEs in the curriculum. In the event of a repeat exam, the overwhelming number of the students who took CBE would choose the CBE again (3.7 on a Likert scale), only a few students (6 out of 36, ca. 15%) stated a preference for PBE in future exams. The PBE-group showed a very high disposition to maintain the PBE-format (4.6). None of the students in this group stated a preference for the CBE format in future tests. In addition, the reason for the choice of the format was evaluated in an open-ended question. The results are presented in table 2.

**Table 2:** Reason for preference

Reason for the preference (frequency of the item) of the computer-based examination format n = 26 of 36	Reason for the preference (frequency of the item) of the paper-based examination format n = 55 of 62
Quick assessment (n = 10, 34%)	Possibility for outlines or written notices on the questionnaire (n = 23, 42%)
Supporting technological advancement (n = 7, 27%)	Better overview (n = 15, 27%)
Support of the computer concerning the number of the items to consider (n = 7, 27%)	Habit (n = 11, 20%)
More objective assessment (n = 2, 8%)	Fear of computer errors or disturbingly loud keyboards (n = 6, 11%)

According to the evaluation of both groups, predominantly no or only very little additional effort was necessary to handle the format, whereas the students of the CBE-group noted higher mental exertion due to the CBE format (CBE: 2.3, PBE: 1.1, p < 0.001). In the CBE-group, the members felt no influence over their performance in the examination, whereas members of the PBE-group perceived an added benefit with respect to their performance in the examination (CBE: 3.0, PBE 4.4; p < 0.001).

Dealing with the examination format was judged to be simple in both groups (CBE: 4.6 vs. PBE: 4.7, not statistically significant), although the CBE was supposed to be more clear and easier to understand (CBE: 4.4 vs. PBE: 4.0, p = 0.12). There were no technical problems in the CBE-group (4.7). In addition, the students in the CBE were very satisfied with the graphical user interface (4.7).

**Figure 1:**
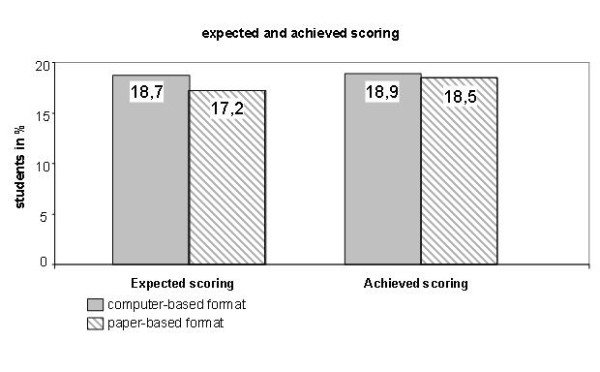
**Expected and achieved scoring of the exam takers**.

**Figure 2:**
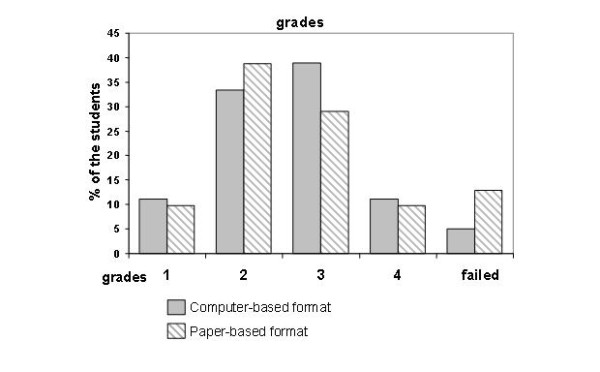
**Distribution of grades in computer-based and paper-based examination**.

There was no difference between the two formats concerning anxiety before (CBE: 3.6 vs. PBE: 3.8, n.s.) as well as the reduction of anxiety after the examination (CBE: 3.5 vs. PBE: 3.2, n.s.). While the students in the CBE-group judged their results in both formats equally, students in the PBE-group had more anxiety of getting poorer results in the computer-based examination. The expected score directly after the examination was higher in the CBE-group, however only 1.5 points in average (total 26 points, CBE: 18.7 points vs. 17.2 points, p < 0.03, see figure 1). The results in both groups were equal, which could be demonstrated in the average number of points (CBE: 18.9 vs. PBE: 18.5, n.s.), discrimination of grades (see figure 2) and the number of students who failed the examination (CBE: 2 vs. PBE: 8, n.s., minimum 15 points).

From the instructors' point of view, the assessment of the computer-based examination was quicker and more efficient (0.5 hours for one instructor for 36 students vs. 60 hours for 62 students). The assessment took 45 min at the CBE for the two open ended questions.

## Discussion

Primary results show a high acceptance of the computer-based examination, which is reflected in a 37% voluntary participation and in a high level of readiness to take further examinations in this examination format. The students of the paper-based examination showed very high willingness to remain in this examination format for upcoming examinations. Additionally, this group showed a high level of anxiety with respect to poorer performance in a computer-based format. On the one hand, this could be a consequence of being used to paper-based examinations. On the other hand, it could be due to additionally stated reasons, which should be taken seriously and diminished in future examinations as effectively as possible (see table 2).

For example, there should be the possibility for written notices on the test (possibility for outlines, personal remarks etc.), including the computer-based examination. This was a motivating reason for 23 students who registered for the paper-based examination. On average, the anxiety before the examination was comparable in both groups. The fear of PC-errors or technical difficulties was nevertheless reason enough for the choice of seven students for the paper-based examination. This anxiety could possibly be lowered by an additional introduction to the examination software or a sample examination for these students. The perceived usefulness of the paper-based examination concerning a personal performance increase in this format could surely be interpreted in the context with the given reasons for the decision to take the paper-based examination. One reason may be the possibility of writing short notices on the test form or a subjectively better overview of the examination can lead to the impression of a higher performance. Moreover, Miller et al. found that the development of visually rich quizzes was greatly facilitated by the use of computers [7]. While Ogilvie et al. demonstrated that students found computer based tests less time consuming [8], we experienced that both groups finished in nearly the same amount of time.

The slightly higher mental exertion required in the computer-based format could be explained by the students' familiarity with paper-based examinations and the rare usage of the graphical user interface. Regular computer-based examinations in the curriculum of the faculty in more subjects with the same examination software could possibly change this impression of the students by familiarizing these students with computer-based examinations and lower the anxiety. On the other hand, the objection of some students concerning disturbingly loud keyboards must be taken seriously (see table 2) and be corrected with the use of special keyboards if the occasion arises.

The examination was judged as simple, there were no technical difficulties, and the students were satisfied with the graphical user interface. The usability was judged to be very high in the CBE-group, especially in clarity and understanding. The students with the CBE judged their examination to be more clear and understandable, however this difference did not reach the level of significance. This is evidence of a high level of security throughout the examination, which is in concordance with other studies [9-12]. Due to the fact that answers to open ended questions could be corrected by only one instructor, the quality criteria of equal treatment could be better achieved and as a consequence objectivity could be raised.

Another essential result of this study is the independence of the exam outcome from the chosen format. In addition, the average score depicted as the distribution of the grades showed no difference. Russell & Haney show that the test results of students accustomed to writing on computer are higher then those written by hand [13]. Despite this, there was a tendency of more students to fail the examination in the PBE-group (8 vs. 2 in the CBE-group, 12.9% vs. 5.6%). However, this is not statistically significant. On the one hand we could not principally rule out a potential bias that the more intellectual students rather chose the CBE, so possible disadvantages due to technical reasons were compensated. On the other hand the better self-assessment of the students in the CBE-group is impressive and may hint towards a more optimistic attitude of these students to support innovation from the beginning.

The main advantage of the computer-based examination is an increase in efficiency and objectivity, because the automatic procession of the examination data is assumed to be less error-prone. Peterson et al. pointed out that an important step in evaluating computer-based examinations is to be sure that the exam format is measuring the examinee's knowledge and not their comfort level or confidence with the technology [14]. Even script concordance tests could be examined computer-based [15]. Some studies showed that a development of a web-based assessment resulted in less administration for course organizers [11,16]. Unfortunately our study design did not allow performing a randomised trial because of legal reasons so we focused on the voluntary aspect. Here we found no differences if the students are free to choose the test format. Since this study was not intended to prove that CBE is equal or superior to PBE for all exam takers, further studies need to test how these results and the evaluation of a computer-based examination (totally or with randomised access) influence the acceptance, the assessment of the usability, and the outcome of the examination, especially in those students who do not prefer a computer-based examination.

With respect to the variety of teaching and examination content, a computer-based examination is not only equally in its efficiency and ability to measure academic performance, but also an instrument to examine applied knowledge and visual skills with the help of innovative questionnaires and the use of complex media and/or new item structures such as the key feature.

## Conclusion

Despite the exam anxiety on the part of the students, 37% chose a computer-based examination format. In total, there were only very few students (ca. 5%) who denied the computer-based option.

We could show that voluntary computer-based examinations lead to equal test scores compared to the paper-based manner. After further improvement and compensation of objectives on the side of the students, a required computer-based format should be of no disadvantage to the students. By providing reliable information and a proper preparation of the students for the exam via an introduction to the software, a CBE could be a good method to conduct written examinations efficiently and fairly.

## Competing interests

The authors report no conflicts of interest. The authors alone are responsible for the content and writing of this article. Authors guarantee that the manuscript has not been published elsewhere and it has not been submitted simultaneously for publication elsewhere. The authors declare that there is no conflict derived by the sources of funding.

## Authors' information

AH specialised in health technology assessment with the focus on evaluation of the impact of Informatics on Medical Education. He evaluated the data and drafted the manuscript. KB took part in the development and the execution of the computer-based examination. AM participated in the design of the study and performed the statistical analysis. JJ is responsible for the medical education program at the Medical hospital and participated in the coordination of the study. All authors read and approved the final manuscript.

## Pre-publication history

The pre-publication history for this paper can be accessed here:

http://www.biomedcentral.com/1472-6920/11/89/prepub
